# Angina and Non-Obstructive Coronary Artery (ANOCA) Patients with Coronary Vasomotor Disorders

**DOI:** 10.3390/life13112190

**Published:** 2023-11-10

**Authors:** Sarena La, Rosanna Tavella, Jing Wu, Sivabaskari Pasupathy, Christopher Zeitz, Matthew Worthley, Ajay Sinhal, Margaret Arstall, John A. Spertus, John F. Beltrame

**Affiliations:** 1 School of Medicine, Faculty of Health Sciences, The University of Adelaide, Adelaide, SA 5000, Australia; sarena.la@adelaide.edu.au (S.L.); rosanna.tavella@adelaide.edu.au (R.T.); jing.wu@adelaide.edu.au (J.W.); sivabaskari.pasupathy@adelaide.edu.au (S.P.); christopher.zeitz@adelaide.edu.au (C.Z.); margaret.arstall@adelaide.edu.au (M.A.); spertusj@umkc.edu (J.A.S.); 2 Central Adelaide Local Health Network, Adelaide, SA 5000, Australia; 3 Basil Hetzel Institute for Translational Health Research, The Queen Elizabeth Hospital, Adelaide, SA 5011, Australia; 4 Southern Adelaide Local Health Network, Adelaide, SA 5042, Australia; ajay.sinhal@sa.gov.au; 5 School of Medicine, Faculty of Health Sciences, Flinders University, Adelaide, SA 5042, Australia; 6 Northern Adelaide Local Health Network, Adelaide, SA 5112, Australia; 7 Saint Luke’s Mid America Heart Institute, Kansas City, MO 64111, USA; 8 School of Medicine, Healthcare Institute for Innovations in Quality, The University of Missouri-Kansas City, Kansas City, MO 64110, USA

**Keywords:** ANOCA, functional angiography, coronary vasomotor disorders, coronary artery spasm, coronary microvascular disease

## Abstract

Angina and Non-Obstructive Coronary Artery (ANOCA) patients often lack a clear explanation for their symptoms, and are frequently discharged with the label of “unspecified chest pain”, despite the availability of functional coronary angiography (provocative spasm and microvascular function testing) to identify potential underlying coronary vasomotor disorders. This study compared the outcomes of ANOCA patients with a coronary vasomotor disorder diagnosis post elective coronary angiography to patients discharged with unspecified chest pain. Using the CADOSA (Coronary Angiogram Database of South Australia) registry, consecutive symptomatic patients (*n* = 7555) from 2012 to 2018 underwent elective angiography; 30% had ANOCA (stenosis <50%). Of this cohort, 9% had documented coronary vasomotor disorders diagnosed, and 91% had unspecified chest pain. Patients with coronary vasomotor disorders were younger and had a similar female prevalence compared with those with unspecified chest pain. New prescriptions of calcium channel blockers and long-acting nitrates were more common for the coronary vasomotor cohort at discharge. In the 3 years following angiography, both groups had similar all-cause mortality rates. However, those with coronary vasomotor disorders had higher rates of emergency department visits for chest pain (39% vs. 15%, *p* < 0.001) and readmissions for chest pain (30% vs. 10%, *p* < 0.001) compared with those with unspecified chest pain. This real-world study emphasizes the importance of identifying high-risk ANOCA patients for personalized management to effectively address their symptoms.

## 1. Introduction

**Figure 1:**
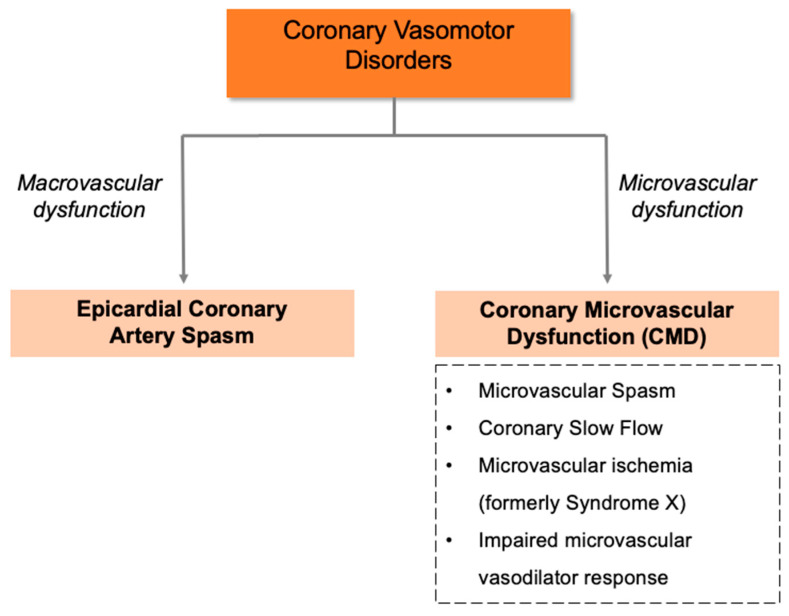
Coronary vasomotor disorder endotypes.

The documentation of coronary artery disease (CAD) through structural coronary angiography provides an explanation for a patient’s presenting symptoms. However, data from the National Cardiovascular Data CathPCI Registry show that almost 60% of patients undergoing elective coronary angiography have non-obstructed coronary arteries (<50% stenosis in any vessel) [1]. These patients are often referred to as ANOCA (Angina with Non-Obstructive Coronary Arteries), as they have no documented explanation for their symptoms, and are frequently discharged with the non-diagnostic label of “unspecified chest pain”. A recent focus group study demonstrated that ANOCA patients frequently experience a complex and long patient journey with suboptimal care, which could be improved with earlier recognition, improved information provision, clear referral pathways, and diagnostic protocols [2]. Functional coronary angiography is the key diagnostic procedure for investigating the underlying pathophysiological mechanisms of ANOCA (Figure 1) thereby providing an explanation for the patient’s symptoms. Hence, ANOCA should be considered a “working diagnosis” in patients with chest pain and non-obstructed coronary arteries, prompting further evaluation of their symptoms.

While conventional diagnostic coronary angiography investigates the intraluminal structure of the epicardial large coronary arteries, functional coronary angiography involves the guidewire-based pathophysiological assessment of the coronary circulation to assess small and large vessel function. This enables the diagnosis of coronary vasomotor disorders in patients with ANOCA (Figure 1). Functional coronary angiography evaluates (1) coronary macrovascular dysfunction via provocative epicardial artery spasm testing with intracoronary acetylcholine (ACh) [3], and (2) coronary microvascular dysfunction (CMD) via the measurement of (i) coronary flow reserve, using thermodilution or doppler wire coronary blood flow measurement of baseline flow relative to the hyperemic response from intravenous adenosine, and (ii) microvascular resistance, using simultaneous coronary pressure and coronary blood flow assessment via doppler (i.e., hMR =hyperaemic microvascular resistance) or thermodilution (i.e., iMR = index of microvascular resistance) techniques [4]. These techniques are readily performed immediately following routine conventional elective coronary angiography, and can more definitively diagnose ANOCA.

The CorMicA trial [5,6] demonstrated that in patients with ANOCA, functional coronary angiography with a stratified medical treatment plan based upon the findings improved 6- and 12-month health status post angiography compared to those who were treated empirically. Consequently, the European Society of Cardiology [7] and, more recently, the American College of Cardiology/American Heart [8] have recommend these techniques in their guidelines. Another study supporting the utility of functional coronary angiography revealed 80–90% of ANOCA patients have abnormal coronary hemodynamic findings [9]. Identifying an underlying coronary mechanism is particularly important, as studies have suggested that ANOCA patients have an increased risk of death or myocardial infarction (MI) compared with the general population [10], and a risk of ongoing chest pain for at least 12 months, similar to individuals with obstructive CAD [11]. More specifically, coronary artery spasm can cause MI, fatal arrythmias, or sudden cardiac death [12,13,14,15], highlighting the importance of appropriate diagnosis. From a patient perspective, functional coronary angiography provides a more appropriate diagnosis than the inappropriate label of “non-cardiac chest pain” in the absence of CAD, and instead provides a patient with better autonomy and assurance [2].

Interest in chest pain investigations that look beyond the structure of coronary arteries is thus rapidly growing. Despite this, functional coronary angiography is seldom undertaken after angiography reveals no obstructed coronary arteries. Communicating an accurate and timely diagnosis to patients is an important component of providing high-quality care [16]. However, diagnostic uncertainty and errors can cause major threats to the quality of care given to patients, and hence there is a clear need for additional research to bring this to light [17], particularly in the setting of ANOCA patients, who are often overlooked.

To highlight the potential to improve care, this study examined real-world insights into the diagnostic approach, management, and three-year outcomes of an ANOCA population. More specifically, the primary objective was to evaluate the prevalence of ANOCA and the use of functional coronary angiography, with a secondary objective to compare the three-year outcomes of ANOCA patients diagnosed with coronary vasomotor disorders with those discharged with a diagnosis of unspecified chest pain.

## 2. Materials and Methods

### 2.1. Patient Enrolment

The Coronary Angiogram Database of South Australia (CADOSA) is a clinical state-wide quality improvement registry of patients undergoing diagnostic coronary angiography and/or percutaneous coronary intervention (PCI). The data specification/definitions are compatible with the American College of Cardiology National Cardiovascular Data Registry (NCDR) [18], specifically the CathPCI Registry [19]. These data definitions are used by all participating CADOSA sites, which includes all public hospitals with a cardiac catheterization laboratory in South Australia: The Queen Elizabeth Hospital, The Lyell McEwin Hospital, The Royal Adelaide Hospital, and Flinders Medical Centre. Hence, the catchment area is the state of South Australia (population of 1.7 million people in 2015). For the duration of this study (2012–2018), there were 30,015 participants on the CADOSA registry. Data are obtained through an opt-out consent approach, allowing comprehensive consecutive data collection. Thus, the participation rate is high, with a total of <1% opting out of the registry.

Trained professional data collectors obtain information through direct patient interviews and hospital records, for which a detailed case report form is completed for each coronary angiogram or PCI procedure. Patient interviews involve obtaining data on symptoms, medical history, and admission medication. Hospital records are used to collect clinical data, including angiographic and procedural details and in-hospital outcomes of consecutive patients. Data on stress or imaging studies performed prior to coronary angiography are also obtained, along with whether the results were negative, positive, or indeterminate. These studies include standard exercise stress test (without imaging), stress echocardiogram, stress testing with SPECT (single-photon emission computed tomography) MPI (myocardial perfusion imaging), and stress testing with CMR (cardiac magnetic resonance imaging). Patients underwent stress testing as per decisions made by the treating cardiologist. The operational management of CADOSA is undertaken by a central management centre, and 5% of the data is routinely audited against the clinical record to ensure data accuracy, with retraining of abstractors if needed.

#### Inclusion and Exclusion Criteria

Patients recruited in the CADOSA registry between January 2012 to December 2018 with the following inclusion criteria were considered to have ANOCA and included in this study (1) were referred for elective invasive coronary angiography for the evaluation of stable suspected ischemic chest pain, and (2) had non-obstructive coronary arteries (i.e., no epicardial coronary stenosis ≥ 50%). The ANOCA cohort was then divided into two groups: (1) coronary vasomotor disorder cohort—i.e., those with a cardiologist-diagnosed coronary vasomotor disorder (coronary artery spasm and/or CMD) confirmed during conventional or functional coronary angiography; and (2) unspecified chest pain cohort—those ANOCA patients without a diagnosed coronary vasomotor disorder.

Coronary vasomotor disorders (coronary artery spasm or CMD) were recorded into CADOSA as reported by the interventional cardiologist. However, additional audit processes within the registry were undertaken to confirm that the conditions were recorded in accordance with appropriate definitions. The definitions for coronary artery spasm diagnosed with acetylcholine during functional angiography include (i) >90% vasoconstriction of epicardial coronary arteries, (ii) ischemic ECG changes, and (iii) chest pain symptoms. Spontaneous coronary artery spasm was identified via conventional coronary angiography through transient narrowing of the epicardial artery. Catheter-induced spasm identified in patients was not considered a coronary artery spasm diagnosis. The definitions for CMD using doppler techniques during functional angiography include a hMR > 1.9 mmHg/cm/s. Coronary slow flow (a CMD endotype) was diagnosed via conventional coronary angiography when there was TIMI-2 (thrombolysis in myocardial infarction) flow grade (≥3 beats to opacify the vessel).

Exclusion criteria included chest pain considered unlikely to be ischemic in nature, such as in those with alternative indications for angiography (pre-operative assessment, valvular heart disease, cardiomyopathy, or an ejection fraction below 40%). Further, individuals presenting with acute coronary syndrome and patients with prior revascularization procedures (PCI/CABG) were also excluded from the study cohort, given the desire to focus on those without known obstructive CAD.

### 2.2. Outcome Data Collection

Data on three-year clinical events including mortality, emergency department presentations for chest pain, hospital inpatient admission, and cardiac procedures were extracted from the South Australian hospital records. The cause and date of death were obtained from administrative records where available. The principal diagnosis for the emergency department presentation and inpatient admission was determined by the principal ICD 10-AM (International Classification of Diseases tenth revision Australian Modification) codes listed on hospital records (Table 1). The cardiac procedures were captured from ACHI (Australian Classification of Health Interventions) codes listed on hospital records (Table 1).

### 2.3. Data Analysis

Patient demographics, clinical characteristics, discharge medications, and three-year outcomes, stratified by coronary vasomotor disorder status, were described as frequencies and percentages for categorial variables, while continuous variables were presented as means and standard deviations. Between-group comparisons were undertaken using independent t-tests for continuous variables and chi-square tests for categorical variables. Linear or logistic regressions were used for age-adjusted *p*-values. Statistical significance was established at an alpha level of 0.05. All statistical analyses were performed using STATA/MP version 17.0 for Windows.

## 3. Results

**Figure 2:**
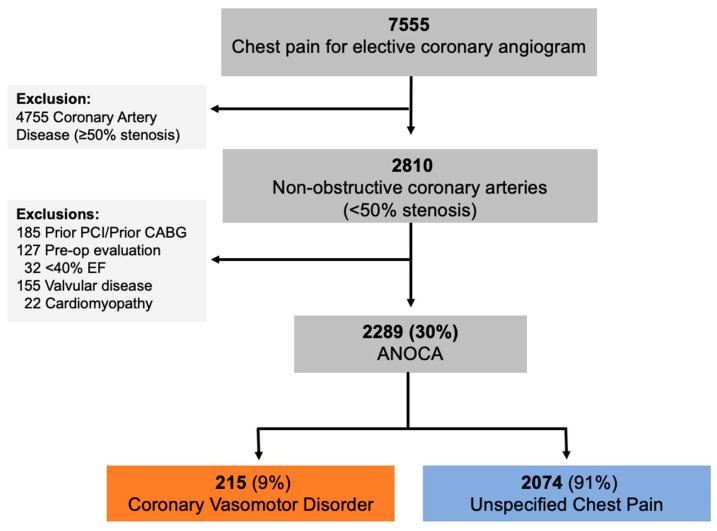
Study selection process for an ANOCA cohort using the CADOSA registry. PCI, percutaneous coronary intervention; CABG, coronary artery bypass grafting; EF, ejection fraction; ANOCA, angina with non-obstructive coronary arteries.

Between January 2012 and December 2018, 7555 patients underwent elective coronary angiography for the evaluation of suspected ischemic chest pain, with 37% (2810) having non-obstructive coronary arteries (Figure 2). Following the exclusion of 511 patients with other indications or prior coronary revascularization, a refined cohort of 2289 patients was identified as having ANOCA. Within the ANOCA cohort, 215 (9%) patients received a confirmed diagnosis of coronary vasomotor disorders, while the remaining ANOCA cases were labeled as “unspecified chest pain” (*n* = 2074) (Figure 2). The discrepancy in numbers of those with a coronary vasomotor disorder diagnosis compared to the number of functional angiograms performed is explained through patients receiving a diagnosis via conventional angiography.

### 3.1. ANOCA Cohort Overview

**Figure 3:**
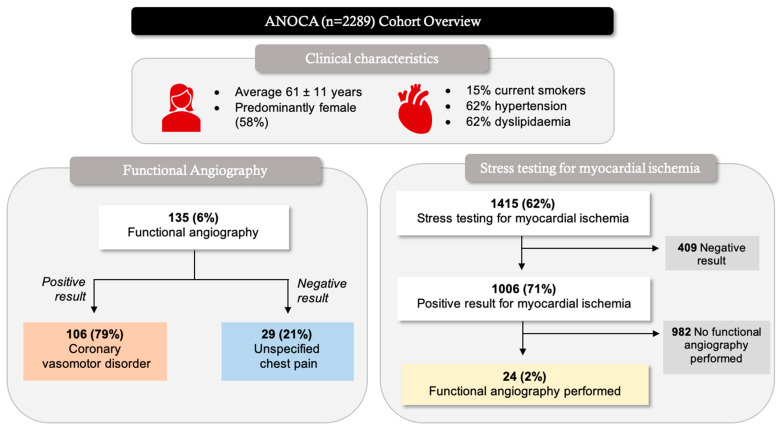
Overview of an ANOCA (*n* = 2289) cohort.

In the overall ANOCA cohort (*n* = 2289), patients were on average 61 ± 11 years, and predominantly female (Figure 3). Functional angiography was undertaken in 135 (6%) of the ANOCA patients, resulting in a 79% positive result for a coronary vasomotor disorder diagnosis (Figure 3). Some patients received a coronary vasomotor disorder diagnosis in the absence of functional angiography. Of the ANOCA patients, 1415 (62%) underwent stress testing, in which 71% had a positive result for myocardial ischemia (Figure 3). Of those with evidence of myocardial ischemia, only 2% underwent further investigation through functional angiography (Figure 3).

### 3.2. Coronary Vasomotor Disorder Breakdown

**Figure 4:**
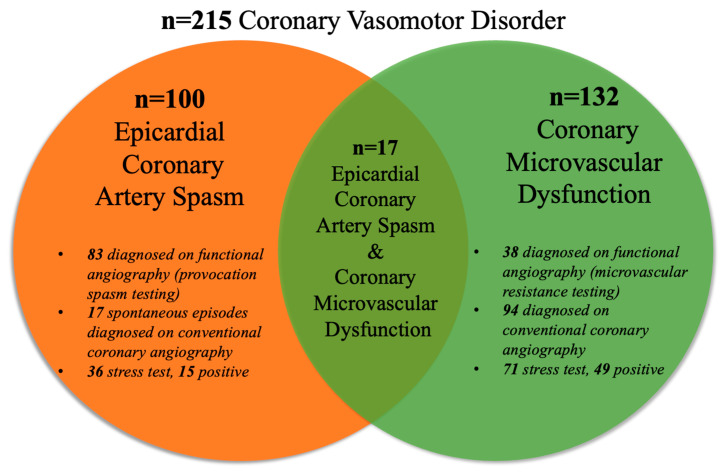
Endotype breakdown in the coronary vasomotor disorder cohort.

Of the 215 patients diagnosed with a coronary vasomotor disorder, 100 had coronary artery spasm and 132 had coronary microvascular dysfunction. Co-existing coronary artery spasm and coronary microvascular dysfunction was diagnosed in 17 patients (Figure 4). Of 100 epicardial coronary artery spasm patients, 83 were diagnosed using functional angiography (invasive spasm provocation, Figure 4). The remaining 17 had spontaneous epicardial coronary artery spasm diagnosed via conventional coronary angiography (Figure 4). Of 132 coronary microvascular dysfunction patients, 38 were diagnosed using functional angiography (coronary microvascular resistance testing, Figure 4). The remaining 94 had coronary slow flow diagnosed via conventional coronary angiography (Figure 4).

### 3.3. Clinical Characteristics, Risk Factors, and Management

Coronary vasomotor disorder patients were significantly younger than the unspecified chest pain cohort, and thus all subsequent analyses were age-adjusted (Table 2). Both cohorts were predominantly female (Table 2). Those with a coronary vasomotor disorder were less likely to have traditional cardiovascular risk factors, such as hypertension and diabetes (Table 2). The coronary vasomotor disorder cohort were more likely to start calcium channel blockers (CCB) and long-acting nitrate therapy at discharge (Table 2).

### 3.4. Three-Year Outcomes

There were no differences between both cohorts in terms of cardiac endpoints, including all-cause mortality, MI, stroke, and heart failure, over 3 years of follow up (Table 3). There were also no differences observed in the rates of repeat coronary angiography.

**Figure 5:**
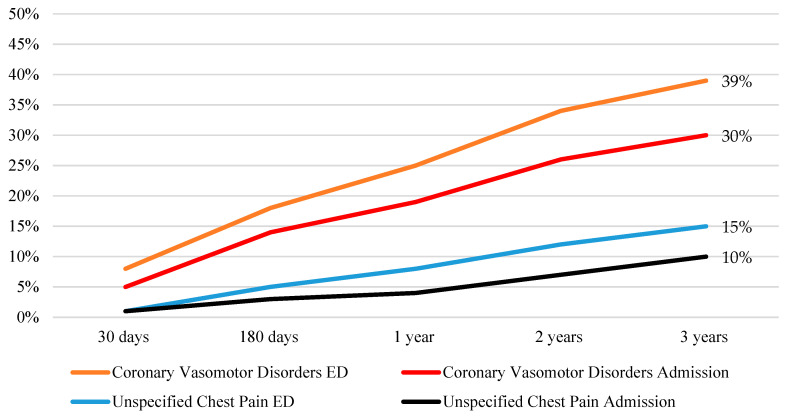
Three-year chest pain presentation in coronary vasomotor disorder and unspecified chest pain cohort.

Those with a coronary vasomotor disorder were more likely to present to the emergency department with chest pain symptoms within three years of their initial angiogram (Figure 5). Similarly, members of the coronary vasomotor disorder cohort were more likely be admitted to the hospital with unstable angina within the 3 years of follow up (Figure 5).

## 4. Discussion

Despite growing awareness of the importance of thoroughly evaluating patients with chest pain undergoing angiography but without obstructive coronary arteries, and emerging guideline recommendations regarding this, the real-world care and outcomes of such patients are incompletely described. In this registry of 7555 consecutive patients undergoing elective coronary angiography for the assessment of suspected ischemic chest pain (angina), the prevalence of ANOCA was 30% (Figure 2). Only 6% of ANOCA patients underwent functional coronary angiography (Figure 3), highlighting the under-investigation and underdiagnosis of these patients. Accordingly, only 9% were diagnosed with a coronary vasomotor disorder, with the remaining 91% being labelled as having “unspecified chest pain” (Figure 2). When investigating outcomes 3 years after angiography, those with a coronary vasomotor disorder diagnosis were more than twice as likely to present to ED with chest pain (39% vs. 15%, *p* < 0.001) and three times more likely to be readmitted due to unstable angina (30% vs. 10%, *p* < 0.001) compared to the unspecified chest pain cohort (Figure 5).

### 4.1. Prevalence

There is heterogeneity in the definitions of ANOCA being used across studies. For example, some studies use a broad criterion for ANOCA such as those with any chest pain in the absence of obstructive blockages on coronary angiography. However, a strict criteria was used for this study to ensure patients were included who were undergoing coronary angiography for chest pain suspected to be ischemic in nature, hence establishing a robust ANOCA cohort. This was achieved by excluding other non-coronary causes for chest pain symptoms, such as valvular disease, cardiomyopathy, and suspected heart failure (defined by <40% EF). Patients undergoing coronary angiography for a pre-operative evaluation were also excluded. Hence, these stringent criteria may explain why the prevalence of ANOCA (30%) in this cohort is slightly lower than previous studies [11].

### 4.2. Diagnosis

In the ANOCA cohort, 215 (9%) had a coronary vasomotor disorder diagnosis, including coronary artery spasm (*n* = 100), CMD (*n* = 132), and co-existing coronary artery spasm and CMD (*n* = 17; Figure 4). This low prevalence does not necessarily indicate a low occurrence of this condition in Australia, but is rather a reflection of the underutilization of functional coronary angiography. Only 6% of the ANOCA cohort had functional coronary angiography testing (Figure 3), highlighting the existing “diagnostic chasm” between patient chest pain burden and the use of pathophysiological evaluations to identify the underlying mechanisms of these symptoms. The low use of functional coronary angiography may be attributed to (a) the limited uptake of the ANOCA concept—despite one of the participating centers being an international leader in this field; (b) the fact that the data collection pre-dates the publication of the CorMICA study; and (c) the fact that regulatory authorities do not provide funding support to undertake this procedure. Contrastingly, in Japan, where there is a high prevalence of vasospastic angina [20], provocative spasm testing is routinely performed during angiography in ANOCA patients, so that many more are identified.

Many of the unspecified chest pain cohort potentially had a missed coronary vasomotor disorder diagnosis, especially considering that in this study, functional coronary angiography had a diagnostic yield of 79% for coronary vasomotor disorder (Figure 3). Other studies showed a similar clinical diagnostic yield using functional coronary angiography in an ANOCA population, ranging from 75% to 90% [21]. Furthermore, within the ANOCA population, 1415 (62%) patients had undergone an exercise stress test, in which 1006 (71%) had a positive result for myocardial ischemia (Figure 3). However, despite having a positive result for myocardial ischemia, only 24 (2%) had undergone functional coronary angiography for further investigation (Figure 3). The common explanation offered is a “false positive exercise test”, which is concerning as this positive result should warrant further investigation into other underlying ischemic mechanisms beyond obstructive CAD. All in all, stress tests demonstrated evidence of ischemia; however, these patients were not provided the opportunity to find explanations for their ischemic symptoms. As is evident in Figure 4, spontaneous epicardial coronary artery spasm episodes were identified via conventional coronary angiography, and hence did not require functional angiography for a diagnosis. Similarly, coronary slow flow diagnosis was detected through conventional coronary angiography via TIMI flow grade.

### 4.3. Clinical Risk Factors

Similar to previous studies on an ANOCA population [5,11], the cohort was, on average, around 60 years and predominantly female (Figure 3). The coronary vasomotor disorder cohort were younger than the unspecified chest pain group; thus, subsequent analyses were age-adjusted (Table 2). Both groups had a similar prevalence of smoking, but the coronary vasomotor disorder cohort was less likely to have other traditional cardiovascular risk factors, such as hypertension and diabetes (Table 2). Coronary vasomotor disorder patients were more likely to have CCB’s initiated at discharge compared to patients with unspecified chest pain (21% vs. 2%, *p* < 0.001; Table 2). Moreover, since CCBs are the first-line therapy in the treatment of coronary artery spasm, they were more frequently prescribed in these patients. In contrast, beta blockers should be avoided in coronary artery spasm but are recommended in CMD patients [22]. This further highlights the importance of delineating the underlying cause in ANOCA to determine appropriate therapy, underscoring the “working diagnosis” approach.

### 4.4. Three-Year Outcomes

Clinical outcomes, including all-cause mortality, stroke, heart failure, and myocardial infarction, were similarly low for both cohorts (Table 3). Although chest pain readmission for unspecified chest pain patients is lower, this may reflect the lack of a formal diagnosis and thus the neglect of symptoms. Indeed, consumer groups report hospital avoidance since they are frequently dismissed as having “nothing wrong with your hearts” given the lack of obstructive CAD. This is also supported by a recent study that evaluated the impact of a definitive diagnosis in an ANOCA population, as some women with an uncertain diagnosis “avoided seeking care altogether” for their symptoms. The women in the study also reported that the risk of the procedure, stress, and time were worth it to receive a definitive diagnosis [2], as it brought a feeling relief and validation and provided a sense of empowerment, whilst giving them greater agency over their bodies [2].

The shortcomings in managing ANOCA patients are well described in Lucy Flanagan’s story, in which an ischemic diagnosis was overlooked in the absence of coronary blockages [23]. Lucy experienced misdiagnosis, numerous tests, and the trialing of a myriad of different therapies which resulted in anxiety, frustration, and a reduced quality of life [23]. After 8 years of suboptimal management, she transitioned from the “unspecified chest pain” to the “coronary vasomotor disorder” diagnosis, following insightful functional coronary angiography [23]. Her coronary vasomotor disorder diagnosis provided a quality of life that Lucy “could previously only have dreamed of”, with greatly reduced symptoms [23]. This story represents the typical journey for ANOCA patients, and is important in highlighting the lack of awareness among clinicians and the clinical impact of functional coronary angiography [24].

## 5. Limitations

This study has significant limitations, including (1) insufficient diagnostic investigation in the unspecified chest pain group, so that it is unclear how many would have had a coronary vasomotor disorder, and (2) a potential selection bias in the coronary vasomotor disorder group, since functional coronary angiography was often reserved for those with suspicious symptoms. These limitations could be addressed in a study in which all consecutive participants underwent functional angiography, and comparisons were made between those with and without a diagnosis. However, these limitations reflect real-world practice and the underutilization of routine functional coronary angiography in Australia.

Additionally, it is difficult to fully appreciate the ongoing chest pain symptoms of both cohorts without patient-reported outcome data, as many patients may have had chest pain symptoms but did not seek acute medical attention. Hence, perhaps an investigation in which Patient Report Outcome Measures (PROMS) were collected and compared with chest pain readmission could highlight this potential avoidance of seeking medical attention in ANOCA patients. The outcome data, including mortality, emergency and hospital admission, and cardiac procedures, were only captured using electronic hospital records that were limited to major South Australian hospitals and a select few rural hospitals. Hence, any events occurring outside of these sites were not captured in the data. Finally, as ANOCA and functional coronary angiography have gained rapid interest in recent years, more recent data are required to reflect the contemporary practices in Australia.

## 6. Conclusions

From this analysis of real-world practice, it can be inferred that few ANOCA patients undergo functional angiography, despite evidence of myocardial ischemia. Consequently, many ANOCA patients are discharged without an appropriate investigation to determine the underlying pathophysiological diagnosis to explain their symptoms. Even when a diagnosis is made, ongoing symptom burden is severe, as over one-third of patients have an unstable chest pain presentation. This study highlights the need to appropriately investigate and treat patients with ANOCA.

## Figures and Tables

**Table 1:** ICD 10-AM and ACHI codes for outcome data collection.

Diagnosis	ICD 10-AM/ACHI Code
Chest pain	R07.3, R07.4, I20.9
Dyspnoea	R06.0
Myocardial infarction	I21.0 to I21.4, I21.9, I22.0, I22.1, I22.8, I22.9
Stroke	G45.9, G46.3, G46.4, I610 to I616, I618, I619, I629 to I636, I638 to I640
Heart failure	I50.0, I50.9
Coronary angiography	38215-00, 38218-00, 38218-01, 38218-02

**Table 2:** Clinical characteristics and new therapies initiated at discharge of coronary vasomotor disorder cohort and unspecified chest pain cohort.

	Coronary Vasomotor Disorders (*n* = 215)		Unspecified Chest Pain (*n* = 2074)		Age-Adjusted *p*
	N	%	N	%	
Baseline characteristics					
Age (mean ± SD, year)	215	57 ± 11	2074	61 ± 11	<0.001
Female	127	61%	1204	58%	0.214
Current smoker	32	15%	308	15%	0.341
Hypertension	110	53%	1311	63%	0.034
Dyslipidemia	114	54%	1302	63%	0.053
Diabetes	27	13%	541	26%	<0.001
Prior MI	10	5%	106	5%	0.995
Cerebrovascular disease	14	7%	135	7%	0.535
PAD	15	7%	88	4%	0.055
Depression	70	34%	617	30%	0.583
Asthma	52	25%	481	23%	0.791
Chronic lung disease	13	6%	220	11%	0.121
Sleep apnea	13	6%	103	5%	0.634
New therapies initiated at discharge					
Anti-platelets	4	2%	21	1%	0.299
Lipid-lowering therapy	8	4%	28	1%	<0.001
ACE inhibitor	3	1%	13	0.6%	0.302
ARB	-	-	8	0.4%	-
CCB	43	21%	37	2%	<0.001
*β* blockers	2	1%	13	0.6%	0.480
Long-acting nitrates	25	12%	25	1%	<0.001

Values are presented as percentages with numbers. Age presented as mean ± SD, year. MI, myocardial infarction; PAD, peripheral arterial disease; ACE, angiotensin-converting enzyme; ARB, Angiotensin II Receptor Blocker; CCB, calcium channel blockers; β, beta.

**Table 3:** Three-year cardiac endpoints of coronary vasomotor disorder and unspecified chest pain cohort.

	Coronary Vasomotor Disorders (*n* = 215)		Unspecified Chest Pain (*n* = 2074)		Age-Adjusted *p*
	N	%	N	%	
All-cause mortality	1	0.5%	38	1.8%	0.301
Myocardial infarction	0	0%	6	0.3%	-
Stroke	3	1.4%	10	0.5%	0.055
Heart failure	1	0.5%	25	1.2%	0.447
Repeat angiography	5	2.4%	53	2.6%	0.976

## Data Availability

The data presented in this study is available on request from the corresponding author. The data is not publicly available due to privacy and ethical restrictions.
